# Significant Reduction of Elevated Triglycerides and Liver Fibrosis in Diabetic Dyslipidemia with Saroglitazar: A Case Report

**DOI:** 10.7759/cureus.6361

**Published:** 2019-12-12

**Authors:** Sayak Roy, Abhijnan Ghosh

**Affiliations:** 1 Internal Medicine, Calcutta Medical Research Institute Hospital, Kolkata, IND; 2 Pharmacology, The Institute of Post-Graduate Medical Education and Research, Kolkata, IND

**Keywords:** hypertriglyceridemia, shear wave elastography, type 2 diabetes mellitus, saroglitazar

## Abstract

Metabolic disorders are characterized by pathologies like visceral adiposity, hypertension, type 2 diabetes mellitus (T2DM), dyslipidemia, impaired glucose tolerance, fatty liver, and so on, with insulin resistance being the main contributing factor. Insulin resistance and diabetes mellitus are commonly associated with elevated triglyceride levels. Among the available medications for treating metabolic disorders, only Saroglitazar has a dual peroxisome proliferator-activated receptor ɑ + γ action that can reduce high triglycerides and improve insulin sensitivity. This medication may also reduce liver fibrosis content. The present case report illustrates the efficacy of Saroglitazar in reducing hypertriglyceridemia and liver stiffness as assessed by shear wave elastography.

## Introduction

In the last three decades, there has been an exponential growth in the incidence of type-2 diabetes mellitus (T2DM) in India. The current prevalence of T2DM in India is 8%-10% of the total population with an additional 15% population having pre-diabetes [1]. Studies have reported that nine out of 10 patients with diabetes are dyslipidemic and that insulin resistance precedes the development of T2DM by decades [2]. Even the pattern of dyslipidemia is different in India as compared to the Caucasian population of the Western world. Low high-density lipoprotein (HDL) is the commonest type of dyslipidemia in India (~72%), followed by high triglycerides (TG) (~30%). In comparison, high low-density lipoprotein (LDL) is the least common type of dyslipidemia (~11%) [3]. Achieving glycemic and lipid goals remains a challenge in the clinical scenario. Saroglitazar is a dual peroxisome proliferator-activated receptor (PPAR) ɑ/γ agonist and is currently available in India for the treatment of diabetic dyslipidemia (DD). It is a potent and predominantly PPAR-ɑ agonist with moderate PPAR-γ agonistic activity. PPARs are nuclear lipid-activated transcription factors that regulate the expression of various genes involved in the control of lipid and lipoprotein metabolism, glucose homeostasis, and inflammatory processes. PPAR-α activation by Saroglitazar increases the hepatic oxidation of fatty acids (FA) and reduces the synthesis and secretion of TG. This, in turn, increases the diversion of FA from peripheral tissues (e.g., skeletal muscle and fat tissue) to the liver. Saroglitazar also activates PPAR-γ and regulates the transcription of insulin-responsive genes involved in the control of glucose production, transport, and utilization. By increasing the expression of these genes, Saroglitazar decreases the post-prandial rise of plasma-free FAs, improves post-absorptive, insulin-mediated suppression of hepatic glucose output, reduces the metabolic burden on liver and muscle, and promotes glucose utilization and insulin sensitivity [4]. Insulin resistance is often observed in nonalcoholic fatty liver disease (NAFLD) [5], which is concomitantly seen in 70% of T2DM patients globally [6]. NAFLD is projected to become the leading cause of liver transplantation, and the risk of liver-related mortality increases exponentially with the increase in fibrosis stage [7]. Currently, no approved drug is available worldwide to treat NAFLD. However, there is ongoing clinical development on various indications of Saroglitazar like nonalcoholic steatohepatitis (NASH; NCT03863574) [8], liver transplant recipients with NAFLD (NCT03639623) [9], NAFLD women with polycystic ovary syndrome (PCOS; NCT03617263) [10], and primary biliary cholangitis (NCT03112681). These trials are in the phase II stage and, based on their outcomes, the future clinical development of Saroglitazar will be decided.

Herein, we report an interesting case of a 26-year-old male patient with diabetes whose severe hypertriglyceridemia was reduced dramatically with treatment using Saroglitazar and statin. The patient also showed a marked reduction in liver fibrosis score as measured by shear wave elastography (SWE).

## Case presentation

A 26-year-old male patient visited the outdoor clinic with his routine workplace-based check-up blood reports of sugar and lipid profiles. On examination, he did not have any signs or symptoms relating to his severely high TG or LDL-c levels. In addition, the patient did not have the financial condition to do any genetic tests to exclude any familial variant or receptor-level mutations. Moreover, he did not have any significant concomitant medical history or family history.

The blood reports showed a high fasting plasma glucose and HbA1c, confirming the diagnosis of diabetes (162 mg/dl and 7.6%, respectively). His lipid parameters were completely deranged with total cholesterol (TC), LDL, and TG being 612 mg/dl, 299 mg/dl, and 2832 mg/dl, respectively. The electrocardiograph and urine albumin-creatinine ratio were normal. By considering his severely deranged lipid profile with strikingly high LDL and TG levels, he was advised to undergo SWE (Affinity 70, Phillips N.V., Amsterdam, Netherlands) to assess the liver fat content and stiffness and obtain the liver fibrosis score. He was put on Saroglitazar 4 mg daily along with Atorvastatin 20 mg. For his T2DM, he was advised lifestyle modifications (LSM) and was asked to re-visit the clinic after three months. However, he re-visited nearly eight months later, with new test reports, owing to job-related issues.

Follow-up

On follow-up, his blood reports showed a marked improvement in lipid parameters along with simultaneous improvements in his blood glucose profile. His fasting plasma glucose (FPG) and glycosylated hemoglobin (HbA1c) were 114 mg/dl and 6.7%, respectively. The striking improvements in lipid profile were reflected by TC, LDL, and TG levels of 122 mg/dl, 65 mg/dl, and 92 mg/dl, respectively. A brief summary of the initial and follow-up data after eight months is described in Table 1.

**Table 1:** Blood glucose and lipid parameters at the 1st visit and the 2nd visit after eight months FPG: Fasting Plasma Glucose; TC: Total Cholesterol; HDL: High-Density Lipoprotein; LDL: Low-Density Lipoprotein; VLDL: Very Low-Density Lipoprotein; TG: Triglyceride; ALT: Alanine Aminotransferase; SWE: Shear Wave Elastography

Laboratory Parameters	Visit 1 (Day 0)	Visit 2 (After 8 months)	Absolute changes from baseline	% change from baseline
Fasting Plasma Glucose (FPG; mg/dl)	162	114	-48	29.63
Glycosylated Hemoglobin (HbA1c; %)	7.6	6.7	-0.9	NA
Total Cholesterol (TC, mg/dl)	612	122	-490	80.07
HDL Cholesterol (mg/dl)	41	39	-2	4.88
LDL Cholesterol (mg/dl)	299	65	-234	78.26
VLDL Cholesterol (mg/dl)	272	18	-254	93.38
Triglycerides (TG, mg/dl)	2832	92	-2740	96.75
Serum ALT (U/L)	74	48	-26	35.14
Shear Wave Elastography (SWE; mean value; m/sec)	1.98	1.59	-0.39	19.70

**Figure 1:**
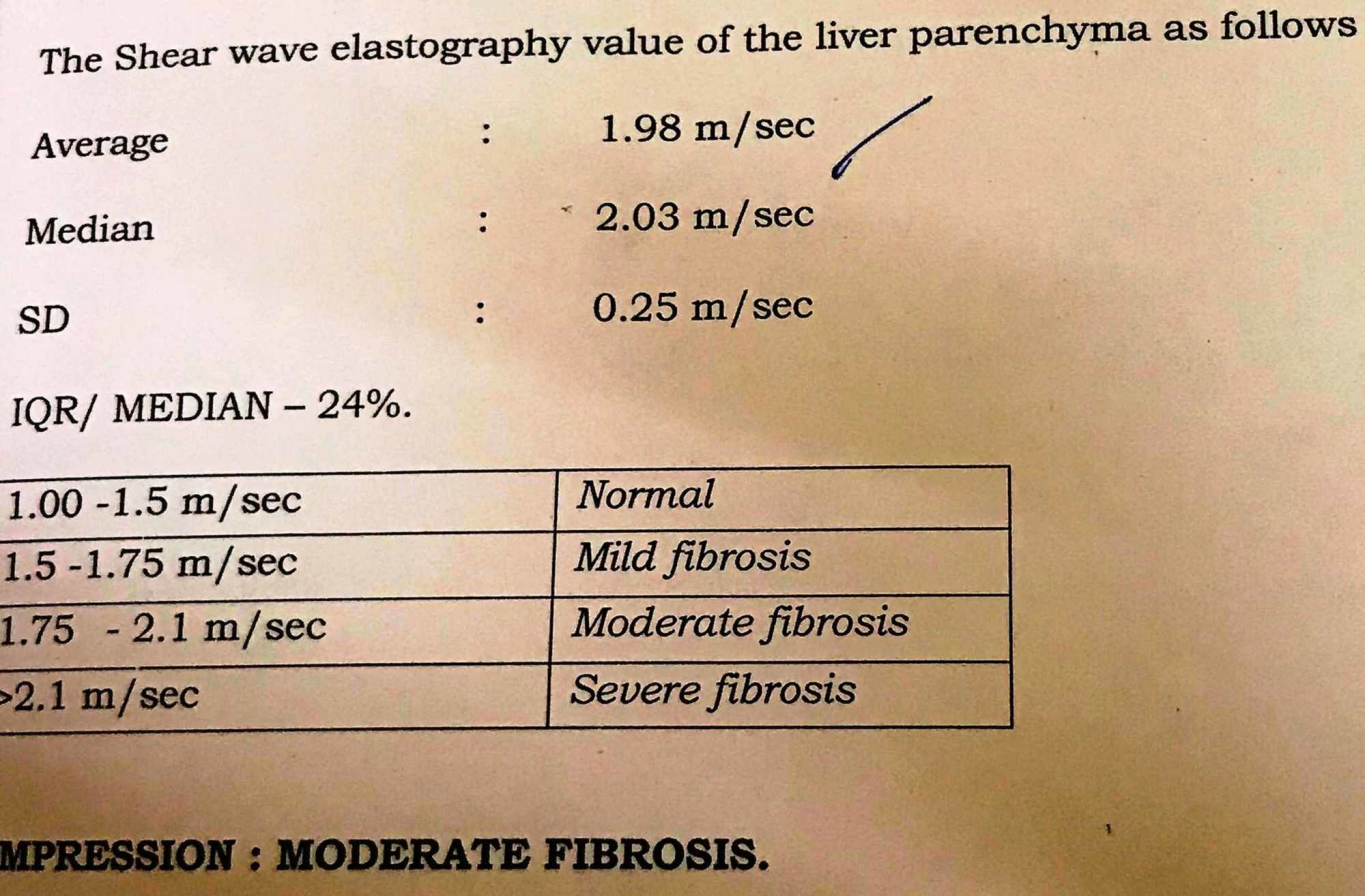
Baseline shearwave elastography value of the patient

**Figure 2:**
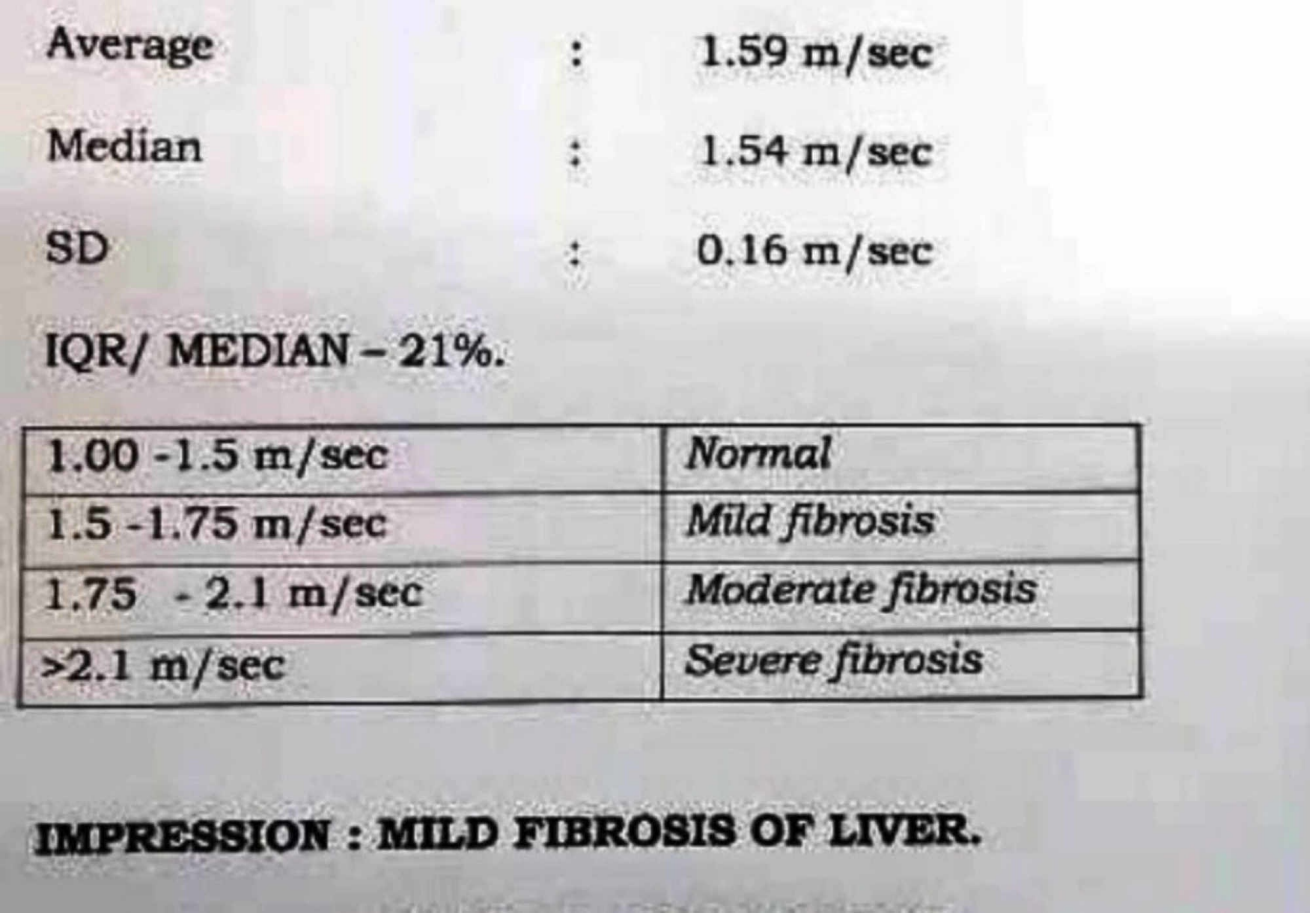
Follow-up shearwave elastography of the patient after eight months

Even the liver fibrosis score came down from 1.98 m/sec (moderate fibrosis as per the acoustic radiation force impulse (ARFI) classification) (Figure 1) to 1.59 m/sec (mild fibrosis as per the ARFI classification) (Figure 2).

The ARFI elastometry method noninvasively quantifies hepatic stiffness and the degree of fibrosis. For example, ARFI values of 1.5 to 1.75 m/s, 1.75 to 2.1 m/s, and >2.1 m/s are considered as mild, moderate, and severe liver fibrosis, respectively [11]. Other parameters that reduced from the baseline were the liver enzymes alanine aminotransferase (ALT) and aspartate aminotransferase (AST). Considering these marked reductions in fasting glucose and lipid parameters, ongoing medications were continued along with LSM.

## Discussion

Insulin resistance is considered to be the core pathology for T2DM and is also associated with dyslipidemia and other metabolic complications such as NAFLD, obesity, and so on. In DD, controlling both glucose and lipid levels appears to be challenging, and a single pill addressing both the entities increases patient compliance to a huge extent. Saroglitazar is the only approved dual PPAR ɑ + γ agonist in India for the treatment of hypertriglyceridemia in T2DM not controlled with statin therapy. Due to its dual action on PPAR receptors, Saroglitazar has a reducing effect on elevated TG and a positive effect on insulin sensitivity. Randomized, controlled, phase 3 clinical trials have shown that Saroglitazar 4 mg once daily when added to statin leads to a significant decrease in TG (−46.7%) and non-HDL- cholesterol (HDL-C; −32.5%) along with a significant decrease in HbA1c (−0.3%). In addition, Saroglitazar was found to be safe and well-tolerated as well [12-13]. A previous study showed that TG was an independent marker of major adverse cardiovascular events in women as reflected by a p-value of 0.01 [14]. In recent studies, TG management has been intensified by the mortality benefits shown in the reduction of cardiovascular events with the icosapent ethyl-intervention trial (REDUCE-IT) study of NCT01492361 with the use of 4 grams per day of icosapent ethyl [15]. The ElastPQ ultrasound shear wave elastography study showed increased TG and diabetes mellitus to be independent risk factors for increased liver fibrosis as measured by SWE (p-value <0.05 for both) [16]. Another study also measured liver stiffness in patients having diabetes using the same SWE technique and showed that 88.88% of the study population comprising NAFLD with diabetes to be above the normal SWE value [17].

In the present case report, the absolute reductions in TC, LDL, and TG levels were -490 mg/dl (80% reduction), -234 mg/dl (78% reduction) and -2740 mg/dl (97% reduction), respectively, post-treatment with atorvastatin 20 mg and Saroglitazar 4 mg for eight months. In addition, his blood glucose parameters were in control with an absolute reduction in FPG and HbA1c by -48 mg/dl and -0.9 % on administering only LSMs and Saroglitazar. Being a PPAR-γ agonist, Saroglitazar has shown improvement in insulin sensitivity in T2DM patients with hypertriglyceridemia [18]. Saroglitazar may have contributed to insulin resistance reduction by acting on PPAR-γ receptors, resulting in improved FPG and HbA1c. In this case study, the results at the eight-month follow-up suggest that Saroglitazar improves lipid parameters as well as glycemic control. This report also highlights the significant reduction in ALT value and SWE score with this molecule, making it a promising future agent in NAFLD with DD.

## Conclusions

Saroglitazar is a dual PPAR ɑ + γ agonist that reduces elevated TG, HbA1c, and stiffness of the liver as reflected by the reduction in the SWE score of the liver. This indicates that Saroglitazar is a dual agonist with triple benefits. Currently, Saroglitazar is under investigation for disorders related to insulin resistance.
